# Prosthodontic Management of a Patient With a Combination of Hemimandibular Hyperplasia and Hemimandibular Elongation: A Clinical Report

**DOI:** 10.1155/crid/1756924

**Published:** 2026-05-06

**Authors:** Mohammed S. Murayshed, Sohil A. Kazim, Steven M. Morgano

**Affiliations:** ^1^ Department of Prosthetic Dental Sciences, College of Dentistry, Prince Sattam bin Abdulaziz University, Al-Kharj, Saudi Arabia, psau.edu.sa; ^2^ Department of Restorative Dentistry, Rutgers School of Dental Medicine, Newark, New Jersey, USA, rutgers.edu

**Keywords:** case report, condylar hyperplasia, full-mouth rehabilitation, hemimandibular elongation, hemimandibular hyperplasia

## Abstract

Hemimandibular hyperplasia and hemimandibular elongation are two distinct forms of condylar hyperplasia that can present clinically with functional and esthetic disturbances. This case report describes prosthodontic treatment of a 59‐year‐old male patient diagnosed with hemimandibular hyperplasia and hemimandibular elongation. It also discusses challenges associated with these anomalies and describes how they were managed in a prosthodontic rehabilitation.

## 1. Introduction

A minor amount of dental and facial asymmetry is frequently observed in patients. Facial asymmetries, however, can occasionally be more pronounced [1]. Hemimandibular abnormalities are assumed to be caused by an abnormal growth pattern of the condyle on one side, and these anomalies have been described as “condylar hyperplasia [2, 3].” However, Obwegeser and Makek [4] maintained that each of these anomalies is distinct and could not be considered simply as condylar hyperplasia. In addition, they postulated the etiology of the two abnormalities. Hemimandibular elongation (HE) and hemimandibular hyperplasia (HH) are two prevalent forms of abnormalities that affect just one side of the mandible and result in pronounced dentofacial asymmetry. These anomalies can be clinically present in a pure form or in a unilateral or bilateral combination [4]. The abnormal growth of the mandible is diagnosed based on the medical history, clinical examination, radiographic findings, and histologic findings. Clinical photos, 2D or 3D imaging, and mounted diagnostic casts can be used to determine the type of growth anomaly.

Single‐photon emission computed tomography (SPECT) scans may be used to assess condylar metabolic activity and are valuable as an adjunct in determining whether condylar hyperplasia is in an active or inactive growth phase, particularly in cases with suspected growth changes [5–8]. Active condylar growth is typically associated with progressive facial asymmetry and occlusal changes, whereas the absence of clinical progression and the presence of a stable occlusion indicate an inactive condition [9–11]. Increased metabolic uptake on SPECT imaging is suggestive of active growth [7, 8]. Nevertheless, SPECT findings should be interpreted in conjunction with clinical evaluation, as symmetrical uptake patterns may not exclude bilateral condylar activity [8].

HH is distinguished by a 3D expansion on the mandible′s affected side. The condyle, condylar neck, ascending, and horizontal rami are all enlarged. These enlargements precisely terminate at the symphysis on the affected side. The clinical manifestations are rotated face, sloping rima oris (lip line cant), and tilted occlusal plane with a possibility of lateral open bite or compensatory downward eruption of the maxillary dentoalveolar segment on the affected side. Furthermore, the mandibular teeth of the unaffected side are tilted lingually, whereas the maxillary teeth on the same side are inclined buccally, creating a scissor bite effect. Those features were first reported in 1836 by Adams, [12] and subsequently further described by others [4, 9, 10, 13]. HE is distinguished by a horizontal displacement of the mandible (unilateral prognathism) and chin to the unaffected side. Intraorally, the affected side exhibits a Class III occlusal relationship with midline shifted toward the unaffected side, and a crossbite relationship is evident. Radiographically, there is a noticeable asymmetry with the body of the mandible elongated in a transverse direction on the affected side. The horizontal rami are on the same level bilaterally [4, 9, 10, 13]. These two conditions differ primarily in the pattern of mandibular overgrowth: HH is characterized by three‐dimensional enlargement involving the condyle, ramus, and body, whereas HE presents predominantly as horizontal overgrowth of the mandibular body, leading to distinct clinical and occlusal features [4].

Management of patients with HH and HE has been previously reported in the literature, and the main course of treatment is focused on orthodontic, surgical, or a combination of orthodontic and surgical treatments [11, 14–16]. The purpose of this clinical report is to describe the prosthodontic management of a patient with a combination of HH and HE, in whom the condition was considered inactive and who required a complete‐mouth rehabilitation. Unique features of this patient were occlusal and esthetic challenges associated with these two conditions, which will be discussed in detail in the following sections.

## 2. Clinical Report

A 59‐year‐old male was transferred to the postgraduate clinics at Rutgers School of Dental Medicine and presented with a chief complaint, “I do not like the way my teeth look, and I want to restore them.” The medical history was noncontributory. Past dental treatment consisted of crowns, direct restorations, endodontic therapy, scaling, and extractions. He stated that his missing teeth were lost because of dental caries. He also stated that he had an acrylic resin mandibular removable partial denture (RPD) with which he was not comfortable. He reported that he is a bruxer. The patient did not report any recent changes in facial appearance or occlusion.

**Figure 1:**
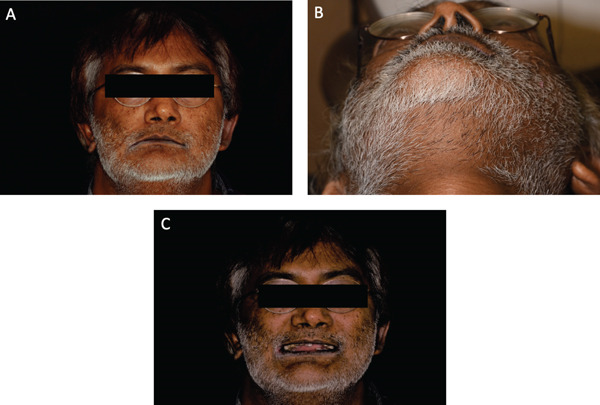
(A–C) Extraoral photos before starting dental treatment.

Extraoral examination revealed facial asymmetry with increased facial height and sloping rima oris on the right side along with chin deviation to the right side (Figure 1A). Viewing the patient from below confirmed the facial asymmetry (Figure 1B). The patient exhibited a square face pattern with a short lower facial third, suggesting overclosure of the vertical dimension of occlusion (VDO). There was a normal range of mandibular movements, with no evidence of joint clicking or mandibular deviation. There was no tenderness on palpation of the TMJs or masticatory muscles [17]. The patient′s VDO, esthetics, and phonetics were evaluated. The interocclusal rest distance was 10 mm with excessive space between his teeth during “S” sound.

Smile analysis indicated a low smile line with inadequate display of maxillary anterior teeth. The maxillary midline was shifted 1 mm to the right of the facial midline. His smile zone showed more teeth on the right side extending to the right maxillary molar, whereas on the left side, the maxillary second premolar was the last visible tooth. There was evidence of a reverse smile line. The patient also presented with signs and symptoms of angular cheilitis (Figure 1C).

**Figure 2:**
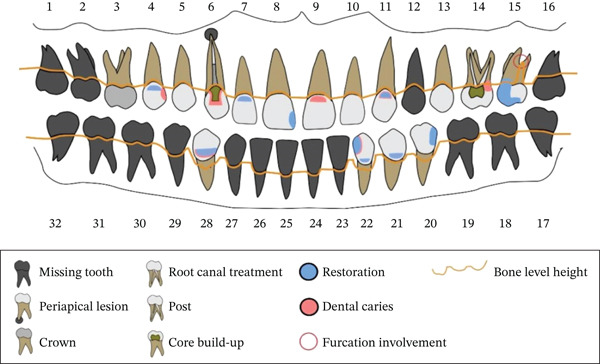
Pretreatment findings.

**Figure 3:**
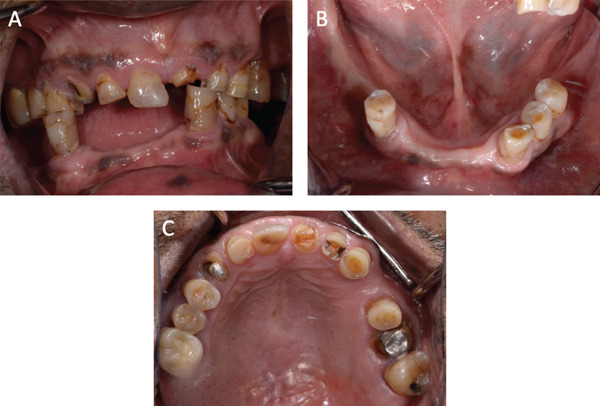
(A–C): Intraoral photos before starting dental treatment.

Examination of teeth indicated that the patient was partially edentulous (Figure 2). He exhibited moderate‐to‐severe occlusal tooth wear in both arches (Figure 3A–C). He also presented with multiple defective restorations and recurrent dental caries. Periodontal examination indicated that there was generalized mild‐to‐moderate clinical attachment loss with an overall stable periodontal condition, except for the maxillary left second molar (Grade III furcation involvement on the disto‐palatal area).

Occlusal examination revealed a Class III angle classification on the left side and Class II on the right side. The occlusal plane on the right side was at a lower level compared with the left side. The mandibular teeth on the left side were inclined lingually, supraerupted, and shifted toward the midline. The mandibular right first premolar was in a crossbite relationship with the opposing teeth. The patient also exhibited inadequate posterior occlusal support (Figure 3A).

**Figure 4:**
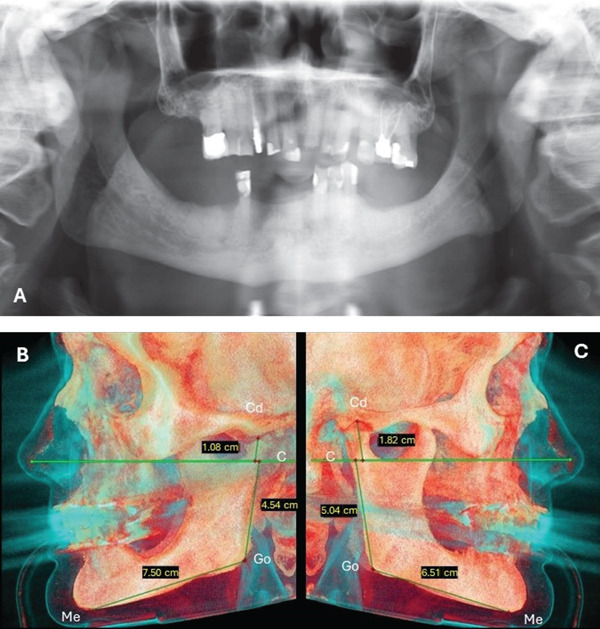
(A) Panoramic radiograph before starting dental treatment. (B, C): Left and right 3D CBCT images with linear measurements, indicating mandibular asymmetry. The following landmarks were used to measure the length of the condyle, ramus, and the body of the mandible: Cd: superior surface of the condyle, C‐point: lowest point of the semilunar incisure, Go: gonion, Me: menton.

A panoramic radiograph indicated mandibular asymmetry. A consultation with a maxillofacial radiologist revealed that the right side exhibited an enlarged and thickened condyle, an elongated ascending ramus, a rounded mandibular angle, and a lowered level of the mandibular border compared with the left side. There was a noticeable coarse and thickened trabecular pattern in the ascending ramus. The left side was associated with an elongation in the body of the mandible. These findings were consistent with the clinical findings of facial esthetics and occlusal disturbances (Figure 4A). A cone‐beam computed tomographic (CBCT) scan of the maxillary and mandibular jaws confirmed the mandibular vertical and horizontal asymmetry. Linear measurements for the condyle, ramus, and body of the mandible were conducted to quantify the mandibular asymmetry as described by Nolte et al. [18] (Figure 4B,C).

Based on these clinical and radiographic findings, the patient was diagnosed with the following:

1. Combination of HH (right side) and HE (left side) in an inactive stage, associated with facial asymmetry, malpositioned teeth, malocclusion, and occlusal cant.
2. Maxillary and mandibular partial edentulism.
3. Localized periodontitis Stage III Grade B (maxillary left second molar), and localized periodontitis Stage II Grade B (mandibular left second premolar, canine, and mandibular right first premolar) [19].
4. Dental caries and defective restorations.
5. Previous root canal treatment of the maxillary right canine with symptomatic apical periodontitis and overinstrumented root canal.
6. Necrotic pulp with normal periapical condition for the maxillary right lateral incisor and the maxillary left second molar.
7. Inadequate posterior occlusal support, accompanied by generalized occlusal wear and loss of VDO (Turner Class I) [20].
8. Probable bruxism [21].
9. High caries risk.
10. Angular cheilitis.

## 3. Treatment Options

**Figure 5:**
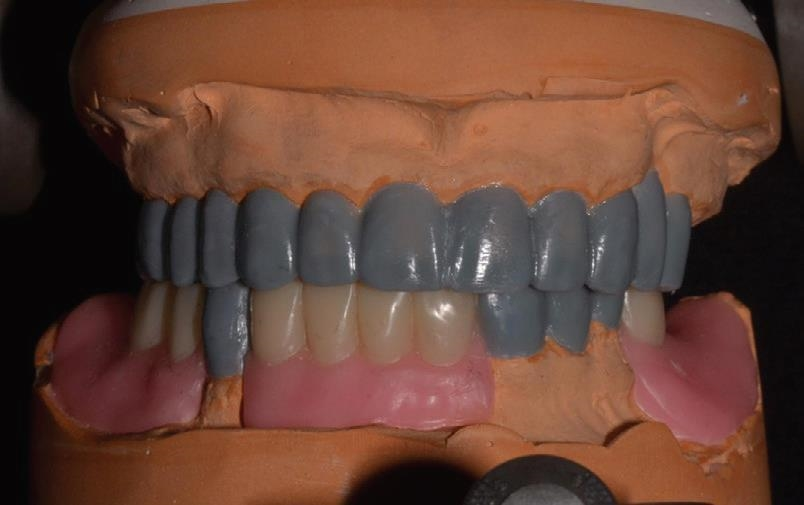
Complete‐mouth waxing for diagnostic purposes.

The incisal edge position of the maxillary central incisors and incisal plane was evaluated clinically. To determine the position of the facial midline, glabella and philtrum were used as reference landmarks because they are generally less likely to present with bilateral asymmetry [16]. Maxillary and mandibular irreversible hydrocolloid impressions were made. The maxillary diagnostic cast was articulated in a semiadjustable articulator by using a facebow transfer record. A centric relation record was made to articulate the mandibular cast. Consultations with oral surgery and orthodontics were performed. The patient declined interdisciplinary treatment because of the time and cost. A diagnostic waxing was made, and it indicated that the occlusal plane and the occlusal scheme could be improved without surgical or orthodontic intervention (Figure 5). The patient understood that prosthodontic treatment alone would not improve his facial asymmetry, chin deviation, or the position of the mandibular teeth. Different prosthodontic treatment options were presented. He opted for conventional metal‐ceramic (MC) crowns and FPDs to restore the maxillary arch and a combination of conventional fixed MC crowns and an implant‐supported RPD to restore the mandibular arch.

A diagnostic mock‐up was made in the patient′s mouth to assist in the diagnostic process. Anterior denture teeth on a record base were evaluated for tooth position, lip support, esthetics, and phonetics. By using the retromolar pads as a reference for the posterior location of the occlusal plane, the diagnostic waxing and the artificial tooth arrangement were completed for the posterior teeth. The casts of the diagnostic waxing without the mandibular interim RPD were duplicated and thermoplastic templates were made and used as a guide for preparation of the teeth for complete crowns. The material was sent to the laboratory to fabricate milled polymethyl methacrylate provisional shells and to process the mandibular interim RPD with a heat polymerized denture base material.

## 4. Treatment

Phase I treatment (disease control) was established to improve oral hygiene and periodontal health. Dietary counseling was conducted, and fluoride (Prevident 5000; Colgate Oral Pharmaceuticals) was prescribed. Also, 0.12% chlorhexidine gluconate (Peridex; 3M Oral Care) oral rinse was prescribed once per day for 7 days per month for caries prevention [22]. Dental scaling and root planing were accomplished. Nystatin–triamcinolone topical cream was prescribed to treat angular cheilitis. All existing restorations were removed, dental caries were excavated and the cavities were restored with composite resin (3M Filtek). The maxillary right canine and left first molar teeth were deemed nonrestorable. Multidisciplinary consultations (radiology, periodontics, endodontics, orthodontics, and maxillofacial surgery) were accomplished.

Phase II treatment was initiated with maxillary and mandibular tooth preparations and provisional restorations at the proposed VDO. The patient wore the interim restorations for 8 months, and he was satisfied with them. At that time, the teeth with an unfavorable prognosis were extracted. After healing, a CBCT scan was made for both arches and indicated the presence of large buccal and mesial bone defects at the site of the maxillary right canine. Therefore, a connective tissue graft followed by an onlay soft graft were performed to prepare the site to receive a pontic with a conventional FPD. The decision to replace the maxillary left first molar was discussed with the patient, and he decided not to replace it with an implant. He reported that he was satisfied with his current esthetics and function [23]. Clinical evaluation revealed that the provisional restorations exhibited adequate and stable posterior occlusal contacts that would ensure long‐term occlusal stability. In addition, extraoral smile analysis of the provisional restorations confirmed that a shortened dental arch would not result in future esthetic problems. Two implants (4 X 9 bone level Neoss ProActive Tapered) were placed at tooth sites of the mandibular right canine and first molar by using a surgical guide. The maxillary left central incisor exhibited a short clinical crown height relative to the proposed incisal edge level of the final crown. Therefore, elective endodontic therapy was performed, followed by a cast post‐and‐core to improve the retentive and resistance form of the tooth preparation. The maxillary right lateral incisor also received endodontic therapy and a cast post‐and‐core.

During Phases III and IV treatment, maxillary and mandibular fixed prostheses were inserted followed by insertion of a mandibular RPD. All existing mandibular teeth were crowned RPD abutments and incorporated specific designs based on the dental surveyor.

**Figure 6:**
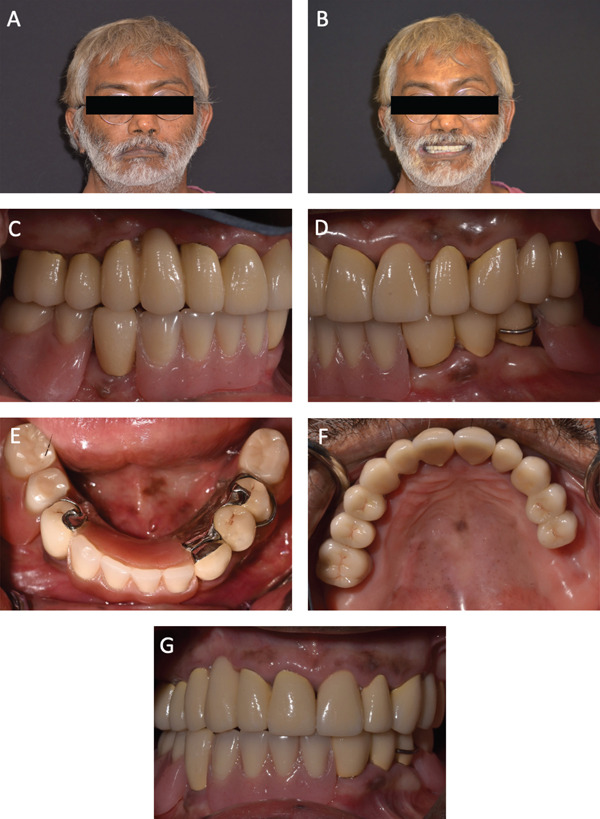
(A–G) Extraoral and intraoral photos after completion of dental treatment.

**Figure 7:**
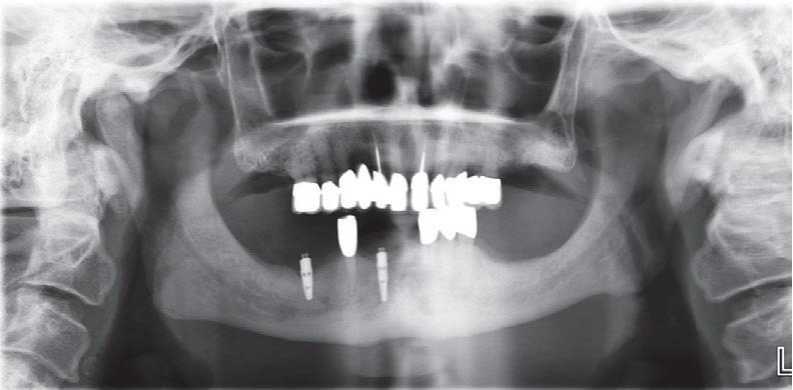
Panoramic radiograph after completion of dental treatment.

Two 3‐mm height Neoss Equator (Neoss) abutments were attached and torqued to the implants as per the manufacturer′s recommendations and metal housings were clinically picked up in the RPD with autopolymerizing acrylic resin (Quick Up–Luting, VOCO). After the metal housings were successfully luted to the denture, the occlusion was evaluated and refined in centric relation and in eccentric movements. The patient was satisfied with the results, particularly with regard to function, esthetics, retention, and stability of the prostheses. (Figures 6A–G and 7). A maxillary occlusal device was fabricated and delivered. Oral hygiene has been continuously reinforced, and he was placed on a regular 4‐month follow‐up to ensure maximal hard and soft tissue health. At the 32‐month follow‐up visit, the patient exhibited a stable oral condition. The prosthodontic restorations also remained in good functional form and relationship.

## 5. Discussion

This clinical report presented a different treatment approach for a case with HH and HE. Thorough clinical and radiographic examinations were performed to assess the magnitude of asymmetry and to determine the types of structures involved [16]. The patient′s complaints and expectations were also carefully evaluated. One study reported that facial asymmetry was not the main complaint for almost one‐third of the patients diagnosed with condylar hyperplasia [9]. For this patient, the facial asymmetry was in both vertical and horizontal planes. However, he was not aware of his asymmetry, nor did he have any signs and symptoms of TMJ dysfunction. If the extraoral esthetics was a major concern for the patient, or if the patient presented with a severe form of occlusal discrepancies, surgical and orthodontic intervention would have been considered as a component of his treatment sequence.

To correct the reverse smile line, the incisal length of the maxillary incisors was increased by using esthetics and phonetics as guides. The proposed incisal plane was evaluated and made parallel to the interpupillary line in the provisional restorations.

The determination of the position and midline of the mandibular anterior teeth was not straightforward because of his mandibular asymmetry. To address this discrepancy, the mandibular anterior artificial teeth were arranged on a record base chairside. It was decided that omitting one incisor tooth would be a suitable option to manage the available space, although the mandibular midline would deviate from the maxillary midline. In addition, because the mandible was deviated to the right, the remaining mandibular teeth were mesially displaced, and the arch length was affected on the patient′s left side. However, because the patient opted not to replace the maxillary left first molar, adding a premolar‐size denture tooth to restore the arch length was not required.

Displacement of the chin and dental arch toward the right side, together with a Class III relationship of the mandibular teeth on the left side, represents clinical manifestations of underlying elongation of the left mandibular body. These occlusal relationships were further accentuated by advanced occlusal tooth wear, loss of posterior support, and the associated reduction in VDO, resulting in an almost edge‐to‐edge anterior incisal relationship and limited restorative space. Although prosthodontic rehabilitation restored facial height and improved the overall maxillomandibular relationship, the individual sagittal tooth relationships remained unchanged, reflecting the pretreatment skeletal asymmetry. In turn, this improvement in jaw relationship allowed the establishment of a functional anterior incisal relationship with appropriate horizontal and vertical overlap to provide effective anterior guidance.

The tilted mandibular occlusal plane was attributed to supraeruption of the teeth on the mandibular left side and the excessive vertical growth of the right side of the mandible, whereby the mandibular right first premolar was at a lower level compared with teeth on the left side. It has been reported that inclined occlusal planes were observed in 48% of patients with active condylar hyperplasia [9]. The discrepancy in the occlusal plane was improved by incorporating more occlusal tooth reduction for the tooth preparations on the mandibular left side. The VDO was increased by 5 mm. With this increase in VDO, the occlusal surface of the mandibular right first premolar could be raised to level the occlusal plane, with a crown‐to‐root ratio of 1:1. Thermoplastic templates were used as a guide to convey these changes to tooth preparations.

Angular cheilitis can affect one or both corners of the mouth. Several local and systemic factors have been linked to this lesion [24, 25]. In the presented patient, poor oral hygiene, *Candida albicans* infection, an ill‐fitting denture, and loss of VDO were assumed to be contributing factors. In addition, angular cheilitis was more evident on the right side as a result of the drooping of the corner of the mouth on that side, and this drooping accentuated deeper commissural folds and provided an environment for microorganisms to reside. Nystatin was prescribed for the short term to address the possibility of fungal infection. However, because the lip line cant was related to the facial asymmetry associated with HH [4, 26, 27], it was felt that complete elimination of the resulting deep commissural folds would not be achieved with the complete‐mouth rehabilitation. Therefore, future recurrence of the infection is a possibility.

To address the crossbite on the right side and lingually positioned and mesially displaced mandibular teeth on the left side, multiple positive occlusal contacts with acceptable horizontal overlap were achieved. The occlusal scheme was also considered for both sides. Because the maxillary and mandibular right canines were missing, partial group function, including both canines and first premolars, was planned to achieve proprioception from natural teeth and yet maintain anterior guidance. Because the mandibular left premolar was in the canine position, the left maxillary canine and mandibular first premolar provided disclusion of the posterior teeth during left laterotrusive movements.

Although some studies have attempted to relate osteoarthritic changes in the condyles to facial asymmetry [28, 29], this relationship is only an association and direct cause and effect has not been established [30, 31]. Therefore, the authors believe that the patient could be safely treated with prosthodontic intervention.

The patient′s age and the assessment of condylar growth activity were important considerations. Although SPECT imaging is considered an important diagnostic tool for evaluating growth activity in condylar hyperplasia, it was not performed in the present case. Condylar hyperplasia is generally regarded as a self‐limiting growth disorder that typically begins during the growth period and becomes inactive after skeletal maturity, although active disease has been reported in patients diagnosed in adulthood [4, 9–11]. In this patient, evidence of long‐standing facial asymmetry, absence of recent progressive skeletal or occlusal changes as reported by the patient, review of previous photographs, and the demonstrated occlusal stability of the provisional restorations over an 8‐month period collectively supported the clinical interpretation that active condylar growth was unlikely.

## 6. Conclusion

Prosthodontic management of patients with mandibular asymmetry may present with some challenges. The course of treatment was determined based on the degree of asymmetry, severity of malocclusion, the patient′s age and growth activity, and the patient′s demands. Because the extraoral facial discrepancy was not the patient′s main concern, this clinical report discussed how challenges associated with asymmetry were managed with prosthodontic treatment.

## Author Contributions

**Conceptualization:** M.S.M. and S.M.M.; **investigation:** M.S.M.; **data curation:** M.S.M. and S.A.K.; **visualization:** S.A.K. and M.S.M.; **writing—original draft:** M.S.M. and S.A.K.; **writing—review and editing:** S.M.M.

## Funding

No funding was received for this manuscript.

## Ethics Statement

Ethical approval was not required for this case report according to institutional policy.

## Consent

Written informed consent was obtained from the patient for publication of this case report and accompanying images.

## Conflicts of Interest

The authors declare no conflicts of interest.

## Data Availability

The data are available from the corresponding author upon request.
